# Anti-Obesity Mechanisms of Plant and Fungal Polysaccharides: The Impact of Structural Diversity

**DOI:** 10.3390/biom15081140

**Published:** 2025-08-07

**Authors:** Guihong Fang, Baolian Li, Li Zhu, Liqian Chen, Juan Xiao, Juncheng Chen

**Affiliations:** 1 Heinz Mehlhorn Academician Workstation, Department of Nutrition and Food Hygiene, School of Public Health, Hainan Academy of Medical Sciences, Hainan Medical University, Haikou 571199, China; fangguihong@hainmc.edu.cn (G.F.); libaolian@hainmc.edu.cn (B.L.); zhuli819@muhn.edu.cn (L.Z.); chenliqian@muhn.edu.cn (L.C.); 2 Medical Devices Research & Testing Center of SCUT, South China University of Technology, Guangzhou 510640, China

**Keywords:** plant polysaccharide, intestinal flora, obesity, mechanism, structure–activity relationship

## Abstract

Obesity, a multifactorial metabolic syndrome driven by genetic–epigenetic crosstalk and environmental determinants, manifests through pathological adipocyte hyperplasia and ectopic lipid deposition. With the limitations of conventional anti-obesity therapies, which are characterized by transient efficacy and adverse pharmacological profiles, the scientific community has intensified efforts to develop plant and fungal polysaccharide therapeutic alternatives. These polysaccharide macromolecules have emerged as promising candidates because of their diverse biological activities and often act as natural prebiotics, exerting beneficial effects through multiple pathways. Plant and fungal polysaccharides can reduce blood glucose levels, alleviate inflammation and oxidative stress, modulate metabolic signaling pathways, inhibit nutrient absorption, and reshape gut microbial composition. These effects have been shown in cellular and animal models and are associated with mechanisms underlying obesity and related metabolic disorders. This review discusses the complexity of obesity and multifaceted role of plant and fungal polysaccharides in alleviating its symptoms and complications. Current knowledge on the anti-obesity properties of plant and fungal polysaccharides is also summarized. We highlight their regulatory effects, potential intervention pathways, and structure–function relationships, thereby providing novel insights into polysaccharide-based strategies for obesity management.

## 1. Introduction

Obesity is becoming a global epidemic affecting all age groups. A recent forecasting study for the Global Burden of Disease Study 2021 indicated that if historical trends continue, the total number of adults living with overweight and obesity will reach 3.80 billion by 2050 (95% uncertainty interval [UI] 3.39–4.04), over half of the estimated global adult population at that time [1]. Regions that will face a significant increase in the number of overweight and obese individuals include Asia and Africa, primarily driven by their growing populations. Overweight and obese children are more likely to remain obese into adulthood and develop non-communicable diseases such as diabetes mellitus (DM) and cardiovascular diseases (CVD) at a younger age [2].

Obesity is also associated with various health issues, including persistent inflammation, oxidative stress, and abnormalities in glucose and lipid metabolism, which subsequently result in an increased incidence of endocrine and metabolic disorders. Psychologically, obesity can lead to lower self-esteem, social isolation, and depression, particularly in children [3]. Common diseases associated with obesity include CVD, sleep apnea, certain types of cancer, type 2 diabetes mellitus (T2DM), hepatic steatosis, and cholesterol gallstones [4,5]. A high-fat diet (HFD) is one of the direct causes of obesity, as it increases calorie intake and impairs insulin sensitivity in the liver. In addition, an HFD activates inflammatory mediators, such as JNK and IKK, thereby promoting hypothalamic inflammation and leading to hypoxia and inflammation in adipose tissue, collectively driving the development of obesity.

Various methods are available for treating and controlling obesity, including weight loss surgeries, dietary adjustments, increased physical activity, and pharmacological treatments [6]. Several drugs are available in the market, such as orlistat, lorcaserin, and liraglutide [7], which can be used to treat obesity; however, they can also cause side effects such as liver and kidney damage and vomiting. In recent years, natural polysaccharides derived from plants and fungi have received growing attention as promising anti-obesity agents due to their high safety, biodegradability, and multifunctional bioactivities. Plant and fungal polysaccharides are macromolecules composed of the same or different aldoses or ketoses connected by glycosidic bonds. They comprise one of the three fundamental substances that make up living organisms, alongside proteins and nucleic acids. Polysaccharides contain multiple functional groups, such as hydroxyl, carboxyl, amino, ester, and sulfate, and therefore have various active sites [8]. Recent studies have shown that their intake can modulate gut microbiota composition and enhance intestinal barrier integrity, thereby mitigating metabolic disturbances associated with obesity cause by an HFD [9].

Some pathogenic mechanisms in digestion and metabolism involve bacterial products and metabolites, including short-chain fatty acids (SCFAs), bile acids (BAs), tryptophan metabolites, and lipopolysaccharides (LPS). These substances influence health by modulating intestinal barrier integrity, metabolic homeostasis, and insulin sensitivity and triggering chronic inflammation [10,11,12]. For instance, polysaccharides from amaranth, buckwheat, and quinoa grains, which are rich in cell wall polysaccharides, have potential applications in functional food formulations [13]. These applications encompass the regulation of gene expression associated with appetite and energy metabolism, restoration of intestinal barrier integrity, reduction of systemic inflammation, and modulation of the gut microbiota composition, thus promoting a transition toward a healthier state. Polysaccharides influence obesity through multifaceted and multichannel mechanisms and exhibit minimal side effects, corroborated by foundational research, animal studies, and clinical trials. In this review, we discussed the complex pathways underlying obesity, focusing on the roles of immune regulation, the nervous system in appetite regulation and energy balance, and the gut microbiota. We explored how these mechanisms contribute to the obesity epidemic and examined the emerging therapeutic strategies targeting these pathways.

## 2. Impact of Plant and Fungal Polysaccharides on Energy Metabolism and Appetite Control

Obesity is characterized by the abnormal accumulation of excess fat in the body. Adipose tissues can be classified into three types: white adipose tissue (WAT), beige adipose tissue, and brown adipose tissue (BAT). Each of these tissues has distinct structural and functional characteristics that contribute to metabolism and energy regulation. The WAT primarily functions as an energy reservoir that stores excess energy in the form of triglycerides. In contrast, the BAT plays a key role in thermoregulation and energy expenditure through non-shivering thermogenesis. Beige adipocytes possess the unique ability to induce thermogenesis, which is closely regulated by uncoupling protein 1 (UCP1). UCP1 enhances energy expenditure by promoting energy dissipation as heat, a process that significantly influences the onset and progression of obesity [14]. In addition to its energy storage function, the WAT regulates energy homeostasis. However, its pathological expansion, whether through hypertrophy or hyperplasia, leads to an imbalance in the tissue microenvironment. This imbalance manifests as chronic inflammation, hypoxic stress, oxidative damage, and metabolic dysregulation, all of which contribute to the development of obesity and related disorders. In addition, this abnormal expansion is associated with structural changes in the tissue, such as impaired angiogenesis and abnormal extracellular matrix remodeling [15].

Plant and fungal polysaccharides can alleviate metabolic disorders through various mechanisms, such as reducing serum cholesterol levels, which in turn helps decrease the incidence of CVD and obesity. For example, seaweed polysaccharides have been shown to enhance thermogenesis by upregulating UCP1/UCP3 expression in the BAT while simultaneously inhibiting lipid accumulation in the WAT [16]. Similarly, pear pomace soluble dietary fiber regulates obesity-related metabolic disorders by inhibiting inflammation and activating the AMPK/PPAR-α signaling pathway, which suppresses the synthesis of anabolic substances such as SREBP-1c and FAS [17]. Polysaccharides from *Padina tetrastromatica* induce thermogenesis and inhibit adipocyte proliferation in obese mice by upregulating UCP1 and downregulating p-Akt, p38, and PPAR-γ expressions in the visceral fat [18]. Another example is the *Taraxacum mongolicum* polysaccharide, which induces brown fat production through the miR-134-3p-mediated AKT/GSK-3β signaling pathway regulation [19]. In addition, polysaccharides derived from *Lyophyllum decastes* have been shown to increase energy expenditure in the BAT of diet-induced obese (DIO) mice [20]. This effect is believed to be mediated by the upregulation of the TGR5 pathway, which is activated by secondary BAs and plays a key role in the anti-obesity effects of these polysaccharides. By altering the gut microbiota and increasing energy expenditure, these polysaccharides significantly reduced obesity and associated metabolic disorders in DIO mice. Collectively, these studies indicate that dietary polysaccharides could serve as a potential therapeutic strategy for obesity, particularly through the activation of thermogenic pathways and the modulation of central appetite control networks.

Appetite regulation is influenced by a combination of genetic, social, and environmental factors involving dynamic interactions between the endocrine, gastrointestinal, and nervous systems. When food enters the duodenum, enteric endocrine cells detect nutrients through specific receptors, triggering transcriptional regulation, the modulation of gastrointestinal motility, and hormone secretion. For example, hormones that promote appetite, such as ghrelin, and those that suppress appetite, including peptide YY (PYY), glucagon-like peptide-1 (GLP-1), gastric inhibitory polypeptide (GIP), cholecystokinin (CCK), amylin, and α-melanocyte-stimulating hormone (α-MSH), act on target organs through paracrine mechanisms, neural signaling, or systemic circulation, ultimately influencing feeding behavior [21]. However, in individuals with obesity, the gut–brain signaling axis is disrupted. Studies have shown that obese individuals exhibit impaired brain responses to nutrients (such as glucose and lipids), which are characterized by blunted neural activity in the hypothalamus and reward circuits upon nutrient ingestion. This deficit may stem from reduced sensitivity to gut-derived hormones (GLP-1 and PYY), insulin resistance, or dysfunctional vagal afferent signaling, leading to attenuated satiety signals and compensatory overeating [22]. The central nervous system (CNS) serves as a key integrative center for peripheral signals and plays a vital role in regulating hunger, satiety, and energy balance [23]. Within the hypothalamic arcuate nucleus, two functionally opposing neuronal populations, neuropeptide Y (NPY)/agouti-related peptide (AgRP) neurons and pro-opiomelanocortin (POMC)/cocaine- and amphetamine-regulated transcript (CART) neurons coordinate energy intake [24]. NPY-AgRP neurons are activated under energy-deficient conditions or by orexigenic signals, such as ghrelin, to stimulate food intake. The CNS integrates signals from autonomic nervous pathways, peripheral hormones (such as leptin [LEP], CCK, GLP-1, and PYY), and circulating nutrients (such as free fatty acids [FFAs]), coordinating multiple brain areas to regulate energy balance and body weight [25,26]. Hypothalamic gliosis and changes in the transcriptome of the hypothalamic arcuate nucleus have also been reported [27]. Roux-en-Y gastric bypass (RYGB), which induces weight loss, reduces glial cell marker expression in the hypothalamic arcuate nucleus, thus indicating that alterations in the CNS may contribute to obesity and its treatment. Recent studies have shown that SCFAs, specifically propionic acid and butyric acid, activate FFAR2/FFAR3 receptors in intestinal L-cells, stimulating PYY secretion and helping prevent and alleviate obesity [28]. Among the adiposity hormones, LEP and adiponectin (ADPN) are crucial for energy homeostasis. LEP secreted by the WAT activates hypothalamic POMC neurons, triggering the release of α-MSH, which then binds to melanocortin-4 receptors in the paraventricular nucleus. This cascade further stimulates the satiety neurons in the lateral parabrachial nucleus, thereby reducing food intake. LEP also enhances sympathetic nervous activity, promoting thermogenesis in the BAT and inhibiting obesity [29,30]. ADPN, the most abundant peptide hormone secreted by adipocytes, improves insulin sensitivity and regulates lipid metabolism by activating AMPK and PPAR-α, ultimately reducing lipogenesis through downstream target molecules [31,32]. A recent study has shown that *tea* polysaccharides can regulate glucose and lipid metabolism by promoting the release of GLP-1 and TGR5 [33]. *Platycodonis Radix* polysaccharides stimulate intestinal L-cells to secrete PYY and GLP-1 by activating FFAR2/FFAR3, thus reducing food intake, enhancing glucose metabolism, and promoting energy expenditure [34]. *Yendo* polysaccharides activate the JAK2/STAT3 signaling pathway, promoting anorectic neuropeptide LEP expression and regulating weight gain in OB/OB mice [35]. Moreover, crude *Gac aril* polysaccharide intervention increases the secretion of GLP-1 and GIP in obese mice, improving insulin sensitivity and regulating energy metabolism [36]. These findings indicate that dietary polysaccharides provide a promising intervention strategy in obesity by activating thermogenesis and regulating appetite through multiple pathways. To provide a clearer overview of these mechanisms, Table 1 summarizes the representative plant polysaccharides, their primary targets, and the physiological effects reported in recent studies.

## 3. Plant and Fungal Polysaccharides Improve BA Metabolism

As final products of cholesterol metabolism, BAs are produced in the liver through enzyme-catalyzed reactions [43]. BAs are classified into primary and secondary based on their origin. Primary BAs, such as cholic acid and chenodeoxycholic acid, are synthesized primarily through the classical (mediated by CYP7A1) and alternative (mediated by CYP27A1 and CYP7B1) pathways. Primary BAs are conjugated with glycine or taurine to form conjugated BAs secreted into the intestine [44]. Approximately 95% of BAs are reabsorbed through enterohepatic circulation, whereas the unabsorbed portion undergoes dehydroxylation by the gut microbiota to form secondary BAs (such as deoxycholic acid). These secondary BAs are then returned to the liver through the portal vein for recirculation [45]. BAs play a critical role in lipid digestion and absorption and regulate various metabolic pathways. By activating nuclear receptors such as the Farnesoid X receptor (FXR) and the G protein-coupled BA receptor TGR5, BAs influence lipid metabolism, insulin signaling, autophagy, and inflammation [46,47]. For example, activation of FXR can suppress lipid synthesis by downregulating gene *SREBP-1c* expression, which in turn reduces the expression of downstream targets such as fatty acid synthase (*FASN*) and acetyl-CoA carboxylase (*ACC*), thereby decreasing fatty acid and triglyceride production and alleviating hepatic lipid accumulation [47]. In addition, FXR promotes fatty acid β-oxidation and triglyceride hydrolysis through the induction of PPAR-α and Ces1 as well as the activation of the AMPK-ACC-CPT1α signaling pathway [48,49,50]. These effects synergistically help clear triglycerides from the liver and support overall lipid metabolism. Furthermore, FXR regulates BA synthesis by inhibiting CYP7A1 and CYP8B1 expressions through the induction of a small heterodimer partner, thus maintaining BA homeostasis [51]. In addition, FXR activation stimulates the release of fibroblast growth factor 15, which plays a role in BA transport and excretion [52]. In contrast, TGR5 activation in the BAT and skeletal muscle can upregulate UCP1, which promotes thermogenesis and energy expenditure, inhibits fat accumulation, and alleviates obesity [53,54]. In addition, activation of TGR5 enhances the secretion of glucagon-like peptide-1 (GLP-1) from enteroendocrine L-cells in the ileum and colon, which improves insulin secretion and glucose uptake, thereby helping to alleviate insulin resistance [45]. TGR5 also plays a role in modulating inflammation by inducing autophagy-related protein expression through the cAMP-PKA pathway. This promotes the shift of macrophages from the pro-inflammatory (M1) to the anti-inflammatory (M2) phenotype, inhibiting the NF-κB pathway and NLRP3 inflammasome activation, ultimately contributing to the preservation of intestinal barrier integrity and reduction of inflammation [55].

**Figure 1:**
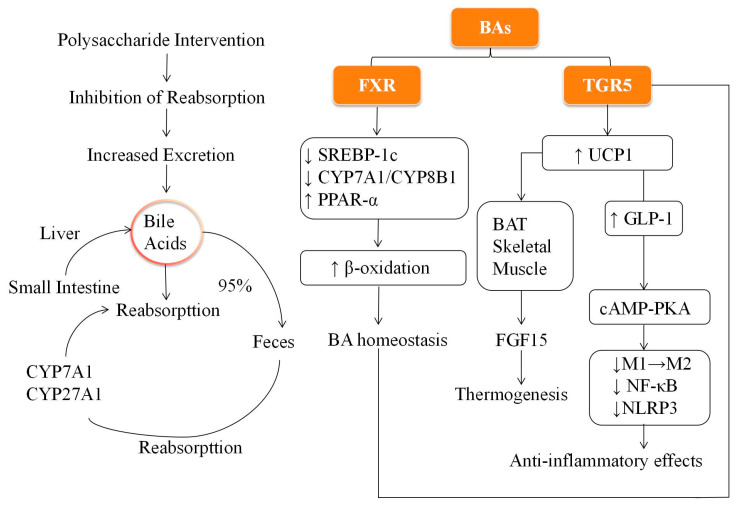
Schematic representation of bile acid metabolism and signaling pathways involved in lipid and glucose regulation.

The presence of polysaccharides in the small intestine can inhibit the reabsorption of BAs, enhance their fecal excretion, and trigger compensatory increases in liver BA synthesis, promoting cholesterol metabolism and lowering serum cholesterol levels [56,57,58]. To facilitate understanding of these interconnected processes, Figure 1 provides a schematic illustration of BA metabolism, FXR/TGR5 signaling, and the modulatory roles of dietary polysaccharides in lipid and glucose homeostasis.

For example, *Pueraria* polysaccharides activated the FXR pathway and promote BA excretion, effectively alleviating diet-induced hyperlipidemia [59]. Similarly, *Astragalus* reduced BA reabsorption in the gut, enhanced liver BA synthesis and excretion, and improved obesity phenotypes in mice [60]. These studies highlight the potential applications of polysaccharide-based natural products for regulating BA metabolism, promoting lipid homeostasis, and combating obesity.

## 4. Plant and Fungi Regulate and Improve Lipid Metabolism

Polysaccharides derived from various plant sources have shown significant potential in regulating lipid metabolism and combating obesity. These bioactive compounds modulate key metabolic pathways involved in lipid breakdown, transport, and storage across different tissues, such as the adipose tissue, liver, and skeletal muscle. The balance between lipolysis and lipogenesis regulates lipid homeostasis. Lipid metabolism involves various processes, including de novo lipogenesis, triglyceride synthesis and hydrolysis, fatty acid oxidation, and adipogenesis [61,62]. The liver, the central organ for both glucose and lipid metabolism, plays a crucial role in regulating these processes. When excessive fatty acids accumulate, they form lipid droplets in hepatocytes, which can interfere with insulin signaling, leading to insulin resistance and promoting obesity [63]. Adipogenesis regulation (adipocyte formation) involves multiple factors. These include peroxisome proliferator-activated receptor gamma (PPAR-γ), adipocyte fatty acid-binding protein 2 (aP2), CCAAT/enhancer-binding protein alpha (C/EBPα), and sirtuin 1, a protein deacetylase that is often upregulated to enhance lipolysis [64,65]. Notably, the activation of Sirtuin 1 is typically linked to an increase in lipolysis. Another important regulator is peroxisome proliferator-activated receptor gamma coactivator 1-alpha (PGC-1α), which promotes gluconeogenesis in the liver by upregulating glucose-6-phosphatase (G6Pase). Inhibition of PGC-1α reduces glucose production and helps lower blood glucose levels [66,67]. In addition, PPAR-α and PPAR-γ both facilitate fatty acid uptake and activation in the liver, helping to reduce hepatic lipid accumulation [68].

In rodent models of HFD-induced obesity and type 2 diabetes (T2D), increased fasting blood glucose levels are accompanied by dyslipidemia, which is characterized by elevated levels of triglycerides (TG), total cholesterol (TC), FFAs, and low-density lipoprotein cholesterol (LDL-C) [69]. Polysaccharides derived from *jackfruit pulp* significantly reduced fasting blood glucose and serum lipid levels in obese mice, possibly through their effects on hepatic lipid synthesis and adipose tissue lipolysis. *Jackfruit* polysaccharides may interact with the insulin signaling pathway, enhancing glucose uptake and promoting lipid oxidation in skeletal muscles [70,71]. Similarly, polysaccharides from *Cordyceps militaris* act by upregulating the expression of genes involved in lipolysis, such as PPAR-α and adipose triglyceride lipase, which facilitate the breakdown of triglycerides in adipocytes. These polysaccharides also inhibit lipogenesis by downregulating the expression of lipogenic genes such as PPAR-γ, SREBP-1c, and FAS, ultimately preventing excessive fat accumulation [72]. Sea buckthorn polysaccharides have been shown to alleviate obesity in mice by regulating the MAPK/ERK and AMPK signaling pathways [73]. AMPK activation promotes fatty acid oxidation and inhibits lipid biosynthesis, whereas MAPK/ERK activation regulates adipocyte differentiation and reduces inflammation in adipose tissues. This dual action highlights the interplay between these two pathways in controlling lipid metabolism and reducing obesity. Furthermore, Chinese yam polysaccharides appear to reduce lipid peroxidation and modulate the expression of proteins involved in lipid metabolism in the liver, such as PPAR-α and SCP-2 [74]. By reducing oxidative stress, these polysaccharides help restore normal lipid homeostasis and mitigate the metabolic disturbances associated with obesity. Hawk tea polysaccharides prevent obesity in mice by increasing the phosphorylation of AMPK and ACC, upregulating carnitine palmitoyltransferase-1 (CPT-1), and downregulating SREBP-1c and FAS expression [75]. The complex interactions between AMPK, MAPK/ERK, and other metabolic pathways in the adipose tissue and liver highlight the intricacy of lipid metabolism regulation in response to these polysaccharides. Moreover, a comparative analysis of the effects of different polysaccharides revealed varying mechanisms of action. Although some target lipolysis and fatty acid oxidation pathways, others primarily reduce lipid biosynthesis and oxidative stress. These variations may inform the development of combined polysaccharide therapies that optimize lipid regulation in obesity management [76].

## 5. Plant and Fungal Polysaccharides Improve Oxidative Stress and Low-Grade Inflammation

Obesity is closely associated with oxidative stress and low-grade inflammation. HFDs can induce oxidative stress, inflammatory responses, and insulin resistance, all of which collectively promote the development of obesity. Inflammation is a protective response to injury or infection, typically linked to elevated endotoxin LPS from Gram-negative bacteria, which triggers pro-inflammatory cytokine secretion and exacerbates inflammation in the adipose tissue [77]. The adipose tissue in obese individuals undergoes changes, producing a variety of inflammatory molecules called adipocytokines. These adipocytokines are involved in the onset of systemic low-grade inflammation, which links obesity and other chronic abnormalities, such as insulin resistance, metabolic syndrome, and CVD [78]. The onset of insulin resistance results from multiple factors, including glucotoxicity induced by reactive oxygen species (ROS), epigenetic changes, activation of transcription factors, and elevated levels of pro-inflammatory cytokines [79]. In individuals with obesity, inflammation-induced insulin resistance is mediated by immune cells, particularly macrophages, which exhibit increased secretion of interleukins, such as IL-1 and IL-6, as well as tumor necrosis factor (TNF)-α [80,81]. These inflammatory factors directly affect intestinal epithelial cells, impairing the expression and function of tight junction proteins (such as claudin-1, occludin, and ZO-1) or triggering inflammatory responses in the adipose tissue, pancreas, and colon [82]. Over time, the resulting chronic inflammatory state results in the accumulation of ectopic lipids in tissues such as the muscle, liver, and blood vessels, further activating local immune cells and promoting the development of organ-specific diseases.

*Ganoderma* polysaccharides can reduce the level of malondialdehyde (MDA) in the serum, while increasing glutathione peroxidase (GSH-PX) and superoxide dismutase (SOD) activities in the liver, thereby improving liver lipid metabolism disorders and effectively inhibiting obesity in mice [83]. In addition, *Ganoderma* polysaccharides can decrease PPAR-γ expression and increase the levels of fatty acids in circulation [84]. In HFD-induced obese rodent models, increased intestinal permeability and decreased expression levels of tight junction proteins, such as claudins-1/3 and ZO-1, were detected. After treatment with specific polysaccharides, such as those from unripe raspberry fruit and longan pulp, significant increases in claudin-1, occludin, and ZO-1 expressions were noted [85,86], as these polysaccharides regulate oxidative stress in the body. In a mouse model of chronic intestinal inflammation induced by an HFD, NF-κB signaling pathway expression and that of its downstream inflammatory genes (such as TNF-α, iNOS, and MCP-1) significantly increased, whereas the levels of inflammation markers (such as CD68, TLR4, TNF-α, iNOS, and MCP-1) in adipose tissue also increased [87]. Furthermore, intervention with *Matsutake* polysaccharides significantly reduced serum levels of TNF-α, IL-1β, and IL-6, as well as the enzyme activities of ALT, AST, ALP, and GGT in the livers of HFD-induced obese mice [68]. Overall, polysaccharides represent a promising approach for counteracting obesity and its associated complications by targeting oxidative stress, inflammation, and metabolic dysfunction. Future research should explore the mechanisms of action and their potential applications in the treatment and prevention of obesity.

## 6. Plant and Fungal Polysaccharides Improve Intestinal Flora Regulation to Alleviate Obesity

The gut microbiota is a complex ecosystem in the gastrointestinal tract that plays a crucial role in physiological processes such as immune regulation, neurotransmitters, and hormone synthesis. Pathogenic bacteria overgrow and the abundance of beneficial bacteria decreases when there is an imbalance in the composition of the gut microbiota, which can damage the intestinal mucosa and increase its permeability. This damaged mucosa releases inflammatory mediators, including cytokines and inflammatory chemicals, which in turn induce systemic or localized inflammation [88]. In the guts of healthy individuals, beneficial bacteria such as *Bifidobacterium* can promote the proliferation and differentiation of regulatory T cells (Tregs), increasing the number of immune cells in the body and helping maintain the immune balance [89]. Furthermore, the gut microbiota can regulate immune responses and affect the production of immune mediators, such as cytokines and immunoglobulins [89]. In addition, the gut microbiota is intricately linked to host metabolism and exerts biological functions through the production of metabolites, providing adequate energy supply and maintaining gut health, immune regulation, and metabolic regulation [90]. The gut microbiota is integral to regulating lipid metabolism in the host, primarily through producing metabolites such as SCFAs and BAs [91]. Studies have shown that *Firmicutes*, such as *Bacteroidetes, Clostridia*, *Lactococcus*, and *Fusobacterium*, produce SCFAs such as butyrate to provide additional energy to the host. The ratio of *Firmicutes* to *Bacteroidetes* (F/B ratio) in the gut microbiota, which comprises 90% of the microbial community, is closely associated with energy homeostasis and metabolic balance regulation. Changes in this ratio can lead to various pathological alterations; for instance, an increased *F/B* ratio is considered a marker of obesity [92]. Individuals with a normal body mass index (BMI) exhibit a greater abundance of Bacteroidetes than obese individuals, who have a higher abundance of Firmicutes [93]. At the genus level, *Faecalibacterium*, *Odoribacter*, *Ruminococcus*, *Coprococcus*, and *Roseburia* are closely associated with obesity and are often enriched in HFD-induced obese mouse models and patients [94,95,96,97,98].

**Figure 2:**
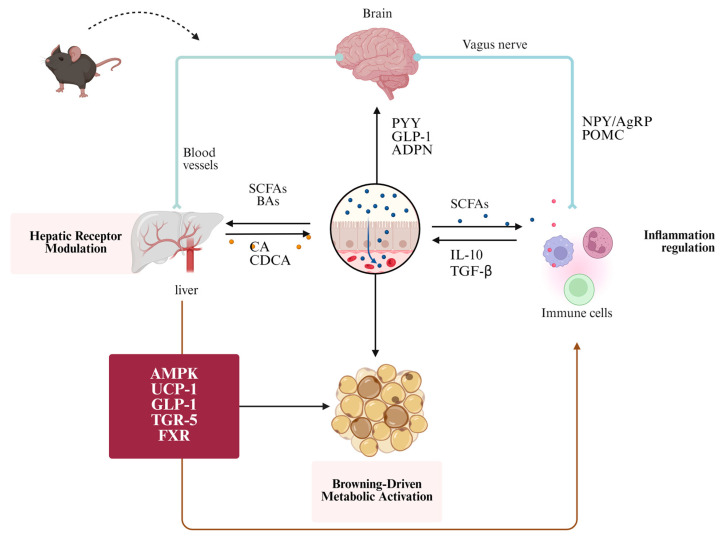
Mechanistic pathways of plant-derived polysaccharides in regulating obesity in animal models.

As signaling molecules, SCFAs exert anti-obesity effects by activating PPAR-γ in the liver and WAT and G protein-coupled receptors GPR43/FFAR2 and GPR41/FFAR3 [99]. SCFAs produced by *Bifidobacterium* and *Lactobacillus* spp. can activate GHSR-1a, which may affect the peripheral nervous system, promote the release of hormones such as LEP and GLP-1, and regulate appetite to promote energy balance [100]. Notably, acetate and propionate can enter the liver through the bloodstream, where they activate energy signaling pathways such as AMP-activated protein kinase (AMPK) in hepatic and muscular tissues, which facilitate glucose uptake and fatty acid oxidation, consequently enhancing blood glucose regulation in murine models [101]. Propionate, produced by gut microbiota such as *Bacteroides*, *Roseburia*, *Firmicutes*, *Ruminococcaceae*, and *Veillonella*, directly affects the satiety signaling of the CNS through GLP-1 and PYY, regulating liver fat synthesis and cholesterol metabolism, thereby inhibiting weight gain [102]. One randomized, double-blind, crossover trial recruited 20 healthy, non-obese male participants to compare the effects of inulin-propionate ester (IPE) and regular inulin on brain responses to food rewards [103]. The results showed that participants who consumed IPE exhibited significantly lower activation in the striatum, particularly in the nucleus accumbens, when viewing images of high-calorie foods, compared to the control group. This indicated that colonic propionate specifically suppressed the reward response of the brain to high-calorie foods. Notably, propionate may reduce cravings for unhealthy foods through a gut–brain axis regulatory mechanism. Some gut microbiota metabolize BAs and conjugated BAs as energy sources and maintain gut and liver glucose tolerance and insulin sensitivity by activating BA receptors such as FXR and TGR5. TGR5 activation regulates GLP-1 production in the gut, thereby improving liver function and glucose tolerance in obese mice [104]. For instance, supplementation with *Lyophyllum decastes* polysaccharides, *Bacteroides intestinalis*, and *Lactobacillus* promotes the increase of secondary bile acids, regulates TGR5 expression, and upregulates energy metabolism-related genes such as PGC-1α, and Ucp1, leading to increased thermogenesis and the alleviation of obesity [105]. Moreover, microalgal polysaccharides have been shown to correct HFD-induced gut ecological imbalance by increasing the abundance of beneficial bacteria (such as *Clostridia*, *Bacteroidia*, and *Mollicutes*) and reducing the abundance of harmful bacteria (such as *Actinobacteria* and *Verrucomicrobia*), thereby restoring normal SCFA and secondary BA metabolism and highlighting the potential anti-obesity mechanisms of the gut microbiota in regulating host lipid metabolism [106]. Following a 9-week intervention with tea mushroom polysaccharides in HFD-fed mice, there was a notable reduction in the relative abundance of *Desulfovibrio* and *Oscillibacter* in the gut microbiota, accompanied by an increase in the relative abundance of *Bacteroides*, *Parabacteroides*, *Butyricimonas*, and *Dubosiella* [107]. Another study reported that *Bacteroides* can hydrolyze conjugated BAs (such as TCA and GCA) into free BAs (such as CA and DCA) through bile salt hydrolase activity, thereby activating FXR. This leads to the inhibition of NLRP3 inflammasome assembly and downstream pro-inflammatory cytokines (such as IL-1β and IL-18), while enhancing intestinal immune homeostasis [108]. SCFAs and BAs produced by the gut microbiota are crucial regulators of metabolism, helping maintain energy balance and influencing liver function. Beneficial bacteria such as *Bacteroides* contribute to immune response regulation and prevention of obesity through their metabolic products. By modulating the composition of the gut microbiota and its metabolic products, polysaccharide intervention helps maintain a dynamic balance between the gut microbiota and host glucose–lipid metabolism. This approach provides novel insights into the prevention and treatment of obesity and related metabolic disorders. To better illustrate the mechanistic and translational relevance of plant-derived polysaccharides in obesity intervention, we categorized the included studies into two groups based on structural clarity. Table 2 presents purified and structurally well-defined plant polysaccharides that have been investigated in animal models for their effects on obesity-related metabolic parameters and gut microbiota composition. These studies allow clearer attribution of functional effects to specific molecular structures. Table 3 presents studies evaluating polysaccharides derived from various plant sources and their effects on obesity-related outcomes in animal models. Figure 2 further shows how these polysaccharides exert anti-obesity effects through multiple mechanisms, including inflammation regulation, lipid metabolism, gut microbiota composition, and appetite.

## 7. Human Clinical Evidence of Plant- and Fungal-Derived Polysaccharides in Obesity

Although preclinical studies in rodent models have provided extensive insights into the anti-obesity mechanisms of plant-derived polysaccharides, including effects on lipid metabolism, insulin sensitivity, and gut microbiota modulation, translational validation in human populations remains essential for evaluating their clinical potential. In recent years, a growing number of randomized controlled trials and meta-analyses have assessed the metabolic effects, safety, and tolerability of these compounds in overweight and obese individuals. Plant-derived polysaccharides, particularly soluble fibers such as β-glucan, glucomannan, inulin, and arabinoxylan, have attracted increasing attention due to their multifaceted physiological activities and favorable safety profiles. Clinical evidence indicates that daily supplementation with plant-derived polysaccharides, generally administered in doses ranging from 2.8–10 g/d for 4–16 weeks, is associated with improvements in body weight regulation, postprandial glucose control, lipid metabolism, and systemic inflammation. Among these polysaccharides, β-glucans sourced from oat and barley have consistently exhibited the ability to attenuate postprandial glucose and insulin responses, reduce low-density lipoprotein (LDL) cholesterol levels, and enhance satiety in overweight and obese individuals. Similarly, glucomannan extracted from konjac has shown efficacy in lowering BMI and fasting insulin concentrations, while also exerting favorable effects on inflammatory biomarkers such as C-reactive protein. Most clinical trials have reported good overall tolerability, with only mild gastrointestinal symptoms observed occasionally at higher intake levels. These findings in humans correspond well with preclinical data derived from rodent models and collectively underscore the translational potential of plant polysaccharides as functional dietary components for the prevention and management of obesity. A selection of representative randomized controlled trials and meta-analyses are summarized in Table 4.

## 8. Factors Influencing the Anti-Obesity Activity of Polysaccharide Structures

Obesity is closely related to the source of polysaccharides and their structural characteristics, particularly molecular weight, monosaccharide composition, glycosidic bonds, functional groups, chain conformation, and spatial configuration. The extraction method for the polysaccharides also significantly affects their properties. For example, in a study of polysaccharides extracted from *Auricularia auricula* using six different extraction methods, the obtained polysaccharides had different physicochemical properties and biological functionalities [141]. Specifically, the ammonium oxalate solution extraction method had a higher extraction rate and polysaccharide content than the other methods investigated, and the polysaccharides from this method showed good scavenging ability against DPPH, hydroxyl, and superoxide anion free radicals and promoted NO production in mouse macrophages.

The effectiveness of different plant polysaccharides can vary significantly depending on their source, structure, and specific biological activities. Another study used fingerprint analysis through high-performance liquid chromatography (HPLC) coupled with chemometrics to characterize and discriminate polysaccharides from different *Ganoderma* spp. [142]. The results showed that polysaccharides from different parts or species of *Ganoderma*, or from the same parts but different geographical regions or strains, can be clearly differentiated. These structural differences can lead to variations in their biological activities, such as immunomodulatory, antioxidant, and antitumor effects, which are relevant to their potential use in obesity management, as obesity is often associated with inflammation and oxidative stress. Consequently, the extraction of polysaccharides from materials with therapeutic potential, coupled with the investigation of their anti-obesity properties, represents a significant strategy for the pursuit of effective obesity treatments.

### 8.1. Molecular Weight (Mw)

The *Mw* of polysaccharides is a key characteristic influencing their physicochemical properties, such as solubility and viscosity, which, in turn, can affect their bioactivity, such as in satiety, gastric emptying, and lipid consumption and excretion. For example, a study of polysaccharides from Glycyrrhiza uralensis obtained using different extraction methods showed that although the polysaccharides had similar monosaccharide compositions and characteristic functional groups, they had different molecular weight distributions, which led to variations in their in vitro antioxidant and immunomodulatory activities [143]. High-molecular-weight polysaccharides may exhibit an enhanced capacity to scavenge free radicals and stimulate cytokine production in dendritic cells compared to their low-molecular-weight counterparts. Notably, high-molecular-weight Cordyceps polysaccharides (100 kDa) have stronger BA-binding abilities than low-molecular-weight polysaccharides [72]. Similar findings have indicated that high-molecular-weight polysaccharides exhibit stronger BA-binding abilities than low-molecular-weight polysaccharides [144]; this effect may be related to the higher viscosity of high-molecular-weight polysaccharides [145]. Compared to low-molecular-weight β-glucans, high-molecular-weight β-glucans help maintain gut homeostasis, promote mucosal regeneration, improve intestinal permeability, and restore intestinal barriers better [146]. Liu et al. showed that 90-kDa mannan effectively reduced lipid accumulation in the liver by modulating PPAR-γ, HSL, and CPT-1 expression [147]. However, excessively high molecular weights may reduce the lipid-regulating activity of polysaccharides because large molecular structures may affect transmembrane permeability, thereby decreasing their lipid-lowering effects. In contrast, low-molecular-weight polysaccharides may not form active polymers such as triple-helix structures [148]. Notably, mannan polysaccharides with a *Mw*s of 70.5 kDa and 133.9 kDa exhibit enhanced BA-binding capacities [149]. Moreover, two polysaccharides (high- and low-molecular-weight) extracted from wheat bran showed different inhibitory effects on α-amylase and α-glucosidase activity. Low-*Mw* polysaccharides exhibited stronger inhibition of enzyme activity, likely because of their smaller molecular size and stronger enzyme-binding affinity, whereas high-molecular-weight polysaccharides showed weaker effects owing to steric hindrance caused by their larger molecular structures [150]. Ma et al. showed that high-molecular-weight natural KGM, owing to its high viscosity and slow movement, may exhibit a more significant effect on the cecum compared to the low-*Mw* KGM-M-1 (147.2 kDa) and KGM-M-2 (21.5 kDa). It is less viscous and can quickly pass through the cecum into the colon and undergo fermentation [151].

Additional studies reinforce this pattern. High-*Mw* polysaccharides, exemplified by *Auricularia auricula-judae* (approximately ~1.2 × 10^6^ Da [130]), form viscous gels that slow gastric emptying and enhance satiety, but their size limits microbial accessibility and subsequent fermentation. Intermediate-*Mw* polysaccharides, such as those from Morchella esculenta (FMP-1, approximately 4.7 × 10^3^ Da; MMP-L, approximately 3.0 × 10^3^ kDa [120,121]), are more readily metabolized by gut microbiota, supporting sustained SCFA production and downstream metabolic benefits. Low-*Mw* fractions, including fucoidan from *Sargassum pallidum* (approximately 5.9–7.3 kDa [122,123]), are rapidly fermented but provide limited viscosity, reducing their capacity to contribute to satiety or bile acid binding. Collectively, current evidence illustrates that molecular weight is not merely a numerical descriptor but a functional axis shaping how polysaccharides exert anti-obesity effects [72,102,104,146,152]. High-*Mw* species tend to enhance satiety and promote bile acid binding due to their increased viscosity. Low-*Mw* fractions are typically more fermentable and elicit rapid microbial responses. Intermediate-*Mw* polysaccharides often exhibit a combination of these characteristics, providing both moderate viscosity and fermentability. However, this emerging concept of an “optimal molecular weight range” should be regarded as a functional hypothesis rather than a fixed threshold. Its relevance is likely context-dependent and influenced by other structural parameters, such as backbone configuration, degree of branching, and chemical substitutions. Future studies should perform standardized molecular weight fractionation alongside in vivo functional assays to refine this model and determine whether molecular weight can serve as a rational design parameter in the development of targeted anti-obesity interventions.

### 8.2. Monosaccharide Composition

The biological activity of polysaccharides is closely linked to their diverse monosaccharide compositions. Most gut barrier-protective polysaccharides are heteropolysaccharides, primarily consisting of monosaccharides such as galactose, mannose, xylose, rhamnose, and arabinose. One study showed that α-1,4-D-galactosiduronic and α-1,5-arabinosidic linkages, especially the former, significantly affected the promotion effect of polysaccharides from *Lycium barbarum* berries on macrophage function [153]. Similarly, pectic polysaccharides from *Rosa roxburghii*, consisting mainly of arabinose (37.2%) and galactose (34.4%) [117,118], were shown to inhibit TLR4 and NF-κB signaling and reduce serum lipids in obese mice, revealing a synergistic role of these sugars in anti-inflammatory and hypolipidemic effects. Galactose has been associated with enhanced SCFA production and the enrichment of butyrate-producing taxa such as *Enterococcus* and *Citrobacter*. For instance, ginseng polysaccharides, composed of >94% glucose but containing pyranose-type galactose residues, modulated gut microbiota to increase SCFA-producing bacteria and reduce inflammation in obese mice [129]. Moreover, galactose and fucose contents have been positively correlated with the suppression of inflammatory mediators and NF-κB signaling pathway regulation [154], consistent with their enrichment in anti-inflammatory polysaccharide structures. Polysaccharides rich in rhamnose and fucose have also shown antimicrobial properties, notably inhibiting the growth of *Staphylococcus aureus* and other harmful strains [155]. Galacturonic acid, often found in pectin-type polysaccharides (such as from sea buckthorn [73,119]), has been shown to enhance lipid regulation through the AMPK pathway. This is consistent with clinical findings showing that apple-derived pectins, which are rich in galacturonic acid and arabinose, can delay gastric emptying and enhance satiety responses in obese adults [140]. These specific linkages may influence the interactions between the polysaccharides and immune cells, affecting their immunomodulatory activity. Polysaccharides from longan pulp consist of rhamnose, arabinose, xylose, glucose, and galactose, which exhibit significant anti-inflammatory effects by reducing pro-inflammatory cytokine levels and inhibiting the iNOS and COX-2 gene expressions in Caco-2 cells, thereby protecting the intestinal barrier [152].

Mannose has been repeatedly linked to improvements in metabolic health. *Sorghum* polysaccharides containing 7.2% mannose activated the PI3K/Akt pathway, improving glucose and lipid metabolism in obese models [156]. Mannan-rich fractions, such as those from *Herba lophatheri* (BLP80-D), have also shown enhanced antioxidant capacity [157]. Mannose supplementation has been reported to suppress weight gain and adipose inflammation in HFD-fed mice, potentially through modulation of gut microbiota and downstream inflammatory pathways [158]. Clinically, konjac glucomannan (composed of mannose and glucose residues) has been associated with reduced BMI and improved satiety in overweight adults, highlighting the translational relevance of mannose-linked structures [139]. Fucose is another functional monosaccharide linked to anti-inflammatory activity. Kelp polysaccharides, characterized by a sulfated α-l-fucose backbone [124], has been shown to reduce serum lipids and improve bile acid metabolism. In addition, the fucose- and rhamnose-rich polysaccharides from *Caulerpa racemosa* improved insulin sensitivity and decreased circulating LPS levels in obese rats [157]. These findings are supported by reports that rhamnose and fucose can inhibit pathogenic bacteria such as *Staphylococcus aureus* and contribute to pro-inflammatory mediator downregulation through NF-κB signaling [159].

The monosaccharide composition of polysaccharides has also been reported to play a crucial role in shaping gut microbiota composition and fermentation behavior. Arabinose is the first to be used by the gut microbiota. It showed a strong positive correlation with beneficial genera, such as *Bacteroides*, *Lactobacillus*, and *Bifidobacterium*, as well as with the production of propionate [159]. For example, *barley grass* polysaccharides enriched in arabinose and xylose, with a well-defined arabinoxylan-type structure, have been shown to reshape gut microbiota composition, reduce systemic inflammation, and ameliorate metabolic dysfunctions in HFD-fed mice [125,126]. This effect may stem from both selective microbial fermentation and downstream modulation of host signaling pathways. Existing clinical evidence further supports these findings. Randomized controlled trials involving arabinoxylan-rich wheat bran supplements have reported significant improvements in insulin sensitivity and reductions in circulating LDL-C levels among overweight individuals [137]. These outcomes indicate that arabinose contributes to microbial ecological shifts as well as exerts systemic metabolic benefits, especially when embedded in complex polysaccharide matrices capable of sustaining targeted fermentation and signaling.

Overall, the biological effects of plant polysaccharides are closely linked to their monosaccharide composition. Arabinose fosters microbial diversity and SCFA production, galactose supports butyrate-producing taxa and intestinal health, fucose and rhamnose enhance anti-inflammatory and antimicrobial functions, and mannose modulates host metabolism through gut microbiota–immune axis interactions. These observations highlight the importance of sugar-specific contributions to therapeutic outcomes and support the targeted design of polysaccharide formulations based on defined monosaccharide profiles.

### 8.3. Glycosidic Bonds

The type of glycosidic bonds plays a crucial role in determining the structure and function of polysaccharides. For instance, polysaccharides from shiitake mushrooms primarily comprise β-(1→3)- and β-(1→6)-linked glucans, which have been extensively documented for their immuno-modulatory activity. These β-glucans interact with innate immune receptors such as Dectin-1 and Toll-like receptors, leading to the activation of macrophages, dendritic cells, and natural killer (NK) cells, thereby enhancing host defense responses [160,161]. In addition to these, polysaccharides with β-1,3 glycosidic bonds and α-Ara*f*-(1→ and→5)-α-Ara*f*-(1→ glycosidic bonds exhibit significant anti-inflammatory effects. For example, polysaccharides derived from *A. auricula* and *longan pulp* have been shown to ameliorate intestinal inflammation by fortifying the intestinal barrier [152]. Further examples can be observed in galactomannans from *Eurotium cristatum*, which possess backbones containing β-D-galactose (Gal*f*) and α-D-mannose (Man*p*) glycosidic bonds. These polysaccharides also have branching chains connected to the primary chain through α-D-mannose glycosidic bonds (1→2 and 1→2,6). The galactomannans have been shown to inhibit the activities of α-amylase and α-glucosidase, improve the composition of the gut microbiota, reduce FAS and SREBP-1c expressions in adipose tissue, promote fatty acid oxidation, reduce fat storage, and inhibit fat synthesis [162]. Similarly, β-1,2 fructan linkages in inulin-type polysaccharides promote SCFA generation and insulin sensitivity through microbiota-dependent mechanisms [112], whereas 1→4 bonds (such as in *Pericarpium Citri Reticulatae Chachiensis*) enable fermentation into beneficial metabolites that improve glucose and lipid metabolism [163]. Moreover, galactose or arabinogalactan oligosaccharides with α-1,6 linkages have relatively strong immunomodulatory activity [164]. Similarly, sea buckthorn berry polysaccharides containing (1→4)-β-D-galacturonic acid residues showed strong immune activities, including lymphocyte proliferation and macrophage activation [165]. In summary, the glycosidic bonds in polysaccharides play a pivotal role in determining their biological activity and therapeutic potential.

Glycosidic bond types are structural motifs as well as key determinants of the biological functions of polysaccharides. Natural polysaccharides derived from sources such as *Lentinus edodes* (*shiitake*), *A. auricula*, longan pulp *E. cristatum*, and *P. Citri Reticulatae Chachiensis* exhibit diverse bioactivities—including immunomodulatory, anti-inflammatory, and anti-obesity effects—that are closely tied to their glycosidic linkage patterns. Specifically, β-(1→3) and β-(1→6) bonds facilitate immune activation; α-(1→6) and β-(1→2) linkages support gut microbiota modulation and SCFA production; and linear or branched structures such as (1→4) or (1→2,6) influence solubility, fermentability, and metabolic enzyme interactions. This structural diversity contributes to the wide variation in anti-obesity efficacy observed across various polysaccharides.

### 8.4. Modification of Polysaccharides and Enhancement of Their Biological Activity

The widespread application of polysaccharides in different fields is largely owing to their abundance, safety, biodegradability, and range of biological functions. However, the inherent biological activities of natural polysaccharides are often inadequate, necessitating structural modifications to enhance their efficacy. Polysaccharides are characterized by the presence of functional groups, such as sulfates, and selenium, which play a role in modulating their bioactivity. The introduction of these functional groups, that is, the chemical modification of polysaccharides, usually changes their *Mw* and conformation, thereby enhancing or endowing them with novel biological activities. Notably, polysaccharides containing high levels of sulfate groups usually exhibit stronger biological activity, and further chemical modifications can effectively alter their properties [166]. For instance, alfalfa polysaccharides (AP) and sulfated alfalfa polysaccharides (SAP) alleviate obesity through distinct mechanisms. AP primarily regulates the colonic metabolic pathway, whereas SAP influences the amino acid metabolic pathway [167]. These differences are reflected in the differential expression of intestinal metabolism-related genes. In addition, the carboxymethylation of persimmon polysaccharides leads to an increase in their molecular weight and a change in their chain conformation, making the modified polysaccharide more easily digested and used by intestinal bacteria [168]. This is supported by a comparative study in which both native and carboxymethylated persimmon polysaccharides were shown to promote the proliferation of *Lactobacillus* strains and modulate gut microbiota composition in vitro. Notably, the carboxymethylated derivative exhibited a stronger bifidogenic effect and enhanced SCFA production, revealing superior prebiotic potential and microbial selectivity after modification. Carboxymethylation and selenization are important chemical modification methods that alter the physicochemical properties of polysaccharides by introducing carboxymethyl (-CH_2_COOH) groups. This modification optimizes the solubility and structural stability of polysaccharides and significantly enhances their biological activities, such as antioxidant, hypoglycemic, and anti-inflammatory effects [169,170,171,172]. After carboxymethylation, *papaya* polysaccharides can transform from having smooth surfaces to porous flake-like structures, while showing significantly enhanced anti-inflammatory activity [173]. Selenylated *Pleurotus eryngii* polysaccharides exhibit reduced molecular weight, more glycosidic bonds, improved thermal stability, and enhanced immune activities, such as enhanced immune factor secretion, bone marrow function, and *macrophage* phagocytosis [174]. Selenylated *Lonicera caerulea* L. fruit polysaccharides showed altered monosaccharide molar ratios and significantly improved antioxidant properties [68]. In addition to the common sulfation [167,175,176], selenization [174,177,178], and carboxymethylation [168,173] modifications, chemical modification with organic chromium–polysaccharide chelates [127,179,179,180] is a promising emerging strategy. Chromium chelation modification may enhance interactions with the insulin signaling pathway by changing the *Mw* and conformation of the polysaccharide and exposing more active functional groups (such as hydroxyl and carboxyl). For example, the *Grifola frondosa* polysaccharide–chromium (III) complex promotes glucose uptake and reduces blood glucose levels by activating the AMPK/GLUT4 pathway [181]. Simultaneously, it inhibits key enzymes involved in liver lipid synthesis (such as FAS and ACC), reduces lipid accumulation, increases the abundance of SCFA-producing bacteria, and reduces the abundance of pro-inflammatory flora (such as *Desulfovibrio*), thereby improving intestinal barrier function and inhibiting systemic inflammation [181]. The *Ganoderma lucidum* polysaccharide–chromium (III) complex activates the intestinal FXR/TGR5 signaling pathway by regulating bacterial metabolites (such as BAs and tryptophan derivatives) to improve lipid metabolism and energy balance [179]. In addition to chemical modifications, physical modifications such as irradiation can also play a significant role in enhancing polysaccharide properties. For instance, irradiation exposes more active functional groups (such as –OH, C=O, and C–H), which may increase the oxidation potential of β-glucan [182]. Notably, γ-irradiation can effectively degrade polysaccharides, such as those derived from *Auricularia polytricha*, by breaking their molecular chains and reducing their *Mw* [183]. This degradation can enhance the biological activities of these polysaccharides, including their anti-hypercholesterolemic effects. Collectively, these results indicate that physical modifications such as γ-irradiation can be as crucial as chemical modifications in improving the functionality of polysaccharides for specific health benefits.

In conclusion, chemical and physical modifications of polysaccharides, such as sulfation, selenization, carboxymethylation, and chromium chelation, are effective strategies to enhance their anti-obesity effects. Each modification targets distinct metabolic pathways, such as immune regulation, lipid metabolism, and gut microbiota modulation, leading to improved obesity management. Sulfation and selenization enhance immune responses and reduce inflammation, whereas carboxymethylation and chromium chelation improve gut health and insulin sensitivity. Furthermore, irradiation represents a promising physical modification that boosts bioactivity and facilitates molecular interactions, contributing to obesity prevention. Continued research into the structure–activity relationship of these modified polysaccharides will provide further insights into their therapeutic potential in treating obesity and related metabolic disorders. With recent advances in research, tailoring the production of polysaccharide derivatives holds significant promise for diverse applications in functional foods, pharmaceuticals, and biomedicine, highlighting the importance of continued innovations in modification techniques to meet specific health and industrial requirements.

## 9. Conclusions and Prospects

Obesity is a complex chronic metabolic disease that threatens the health of patients and poses a significant economic burden to the public health system. Patients with obesity are more likely to develop other metabolic disorders, such as T2D, non-alcoholic fatty liver disease, and CVD. Plant polysaccharides have attracted significant interest because of their health benefits and excellent biocompatibility. A growing body of research evidence has shown that plant polysaccharides regulate blood glucose levels through various mechanisms, exhibiting antioxidant and anti-inflammatory effects and influencing signaling pathway regulation, glucose uptake inhibition, and gut microbiota modulation. These mechanisms have been confirmed in cell and animal models, showing the potential of plant polysaccharides as anti-obesity agents. The anti-obesity activity of plant polysaccharides may be closely related to their *Mw*, component distribution, degree of branching, and chain conformation. To fully realize their potential, it is necessary to prepare high-purity, structurally clear, and homogeneous plant polysaccharides.

In addition, the complex interactions between plant polysaccharides and gut microbiota remain an important yet underexplored dimension. These interactions involve shifts in microbial composition as well as alterations in microbial metabolites that affect host energy homeostasis, lipid metabolism, and systemic inflammation. However, advancing our understanding of these biological effects faces a critical barrier: the inherent complexity and variability of polysaccharide structures. These macromolecules often exhibit significant heterogeneity, and their composition, molecular conformation, and bioactivity are influenced by factors such as plant species, cultivation environment, harvesting time, and extraction procedures. Such variability can lead to inconsistencies between batches and complicate the reproducibility of biological effects across studies. Furthermore, studies often rely on crude or partially purified preparations, making it difficult to attribute observed metabolic effects to specific polysaccharide components. Without rigorous chemical characterization, including detailed analysis of molecular weight, monosaccharide composition, and glycosidic linkages, it is challenging to clarify structure and function relationships, to identify consistent biological targets, or to establish optimized dosing strategies. Overcoming these obstacles is essential for the development of effective and standardized polysaccharide-based interventions for obesity management. Despite progress in preclinical studies, most investigations on plant polysaccharides have been limited to in vitro systems or animal models, which may not fully reflect their physiological effects in humans. Bridging this translational gap requires more comprehensive exploration of structure–activity relationships in combination with well-designed clinical studies. In particular, human trials are required to evaluate the therapeutic efficacy as well as the safety and tolerability of these compounds. Although plant polysaccharides are generally regarded as low-toxicity agents owing to their natural origin, systematic assessments are required to confirm their safety profiles under different usage scenarios. Such evidence will be critical for advancing the clinical application of plant polysaccharides in obesity management.

Safety has been evaluated using various means in studies on natural products and drugs. For example, in a study on the safety, tolerability, pharmacokinetics, and pharmacodynamics of fimasartan in healthy subjects, safety was assessed by monitoring adverse events as well as changes in vital signs and laboratory parameters [184]. Fimasartan was noted to be safe and well-tolerated; however, it showed an increased incidence of low blood pressure and postural dizziness at a dose of 360 mg after repeated administration. Therefore, similar comprehensive evaluations of plant polysaccharides are required. These evaluations include assessing potential side effects, such as gastrointestinal disturbances, allergic reactions, and any impact on organ function. In addition, long-term safety studies are necessary because using plant polysaccharides for obesity management may require continuous intake over extended periods.

Considering that chemical modifications may significantly influence the biological activity of polysaccharides, future research should explore methods to optimize the anti-obesity effects of plant polysaccharides through chemical modifications. Through these efforts, safer and more effective plant polysaccharides can be developed to address the growing global obesity problem.

## Figures and Tables

**Table 1:** Mechanistic pathways through which plant polysaccharides regulate appetite and energy balance.

Polysaccharide Source	Mechanism	Target Pathway	Physiological Effect	Reference
*Tea* polysaccharides	Stimulate GLP-1 and TGR5 secretion	Incretin pathway	Improve glucose and lipid metabolism	[33]
*Platycodonis Radix* polysaccharides	Activate FFAR2/FFAR3 in L-cells	PYY and GLP-1 release, gut–brain axis	Reduce food intake and increase energy expenditure	[34]
*Yendo* polysaccharides	Activate JAK2/STAT3 signaling	Upregulate LEP (leptin)	Suppress appetite and control weight gain	[35]
*Gac aril* crude polysaccharides	Increase GLP-1 and GIP secretion	Enhance incretin signaling	Improve insulin sensitivity and energy metabolism	[36]
Inulin-type fructans	Fermentable fiber increases SCFAs; enhances GLP-1 and PYY secretion	FFAR2/FFAR3–incretin pathway	Enhanced satiety; improved insulin sensitivity	[37]
*Tremella fuciformis* polysaccharides	Modulate gut microbiota composition; increase SCFAs; enhance GLP-1/PYY secretion; reduce inflammation and adiposity by microbe–gut–brain axis	Firmicutes/Bacteroidetes ratio; SCFA–FFAR2 axis	Reduced weight gain; improved glucose and lipid metabolism; decreased inflammation	[38]
*Mulberry leaf* polysaccharides	Modulate gut microbiota; induce browning of iWAT; activate BAT thermogenesis	Microbiota remodeling and thermogenic activation	Reduction (20–50%) in weight gain; improved lipid profile	[39]
*Pear pomace* soluble dietary fiber	Activates adiponectin (ADPN); stimulates AMPK and PPAR-α signaling; reduces inflammation	ADPN-AMPK/PPAR-α pathway	Suppressed fat deposition; reduced adipose inflammation in HFD-fed mice	[17]
*Platycodonis radix* polysaccharides	Modulate gut microbiota and metabolites; increase SCFAs; enhance PYY secretion	Microbiota–SCFA–PYY– gut–brain axis	Suppressed weight gain; improved glucose lipid metabolism; enhanced satiety and energy balance	[34]
*Tibetan Brassica rapa* L. polysaccharides	Modulate lipid synthesis enzymes (HMGCR, CYP7A1, PPARγ, ACC, FAS, SREBP-1c); restore gut barrier; increase SCFAs through microbiota modulation	Lipid metabolism signaling	Reduced TC, TG, LDL-C; improved lipid profile and hepatic adipose tissue morphology	[40]
*Artemisia sphaerocephala Krasch polysaccharides*	Shape gut microbiota; elevate SCFAs and succinate	Induce UCP1-mediated adipose thermogenesis	Promote fat browning and reduce obesity	[41]
*Fucoidan*	Modulates gut microbiota and increases SCFA levels	Activates thermogenic genes in adipose tissue (such as UCP1)	Enhances energy expenditure and reduces adiposity	[42]

**Table 2:** Structurally defined plant-derived polysaccharides with anti-obesity effects.

Polysaccharide Source	Mouse Model	Dose	Mechanism	Key Effects	Reference
Arabinoxylan (Wheat)	C57BL/6J	7.5% diet (~15 g/day), 8 weeks	↑ SCFA (propionate); ↑ Bacteroidetes/Roseburia; ↑ tight junction proteins	↓ fat mass, ↓ inflammation; improved glucose and lipid profile	[109]
Arabinoxylan (Rice bran)	HFD-fed C57BL/6J mice	5–10 g/kg·d, 8 weeks	Restored α-diversity; ↑ Akkermansia/Bifidobacterium; ↓ TNFα & IL-6	↓ body weight gain; ↓ lipid accumulation	[110]
Arabinoxylan+ Green tea	HFD-fed mice	8 weeks	modulated microbiota differently; increased SCFA	↓ fat mass; improved lipid/glucose parameters	[111]
Inulin	HFD-fed C57BL	16 weeks	↑ SCFAs; ↑ bile acid excretion; ↓ SREBP-1c; ↓ hepatic TG synthesis	↓ BW gain; ↓ TG/LDL; ↑ insulin sensitivity	[112]
Inulin (chicory root) different degrees of polymerization	HFD-fed Sprague Dawley rats	1 g/kg·d, 3 weeks	DP-specific: longer chains modulate microbiota; SCFA production; ↓ glucose	DP27 reduced weight gain; ↓ glucose	[113]
Konjac Glucomannan	HFD-fed C57BL/6J mice	8% *w*/*w* diet (8 g/kg·d), 10 weeks	↑ β_3_-adrenergic receptor (ADR3β) → ↑ UCP1 thermogenesis in iWAT; modulation of lipid metabolism genes	↓ body weight; ↓ adipose accumulation; ↓ plasma lipids; improved glucose tolerance	[114]
Xyloglucan Oligosaccharides (Tamarind seed)	HFD-fed C57BL/6J mice	4.8 g/kg/day, 19 weeks	Modulated gut microbiota (↑ *B. pseudolongum*, ↓ *Klebsiella* spp.); regulated lipid metabolism via gut–liver axis; suppressed systemic inflammation	↓ body weight gain (~12.8–23.3%); ↓ liver steatosis; ↑ microbial diversity; ↓ inflammatory markers	[115]
β-glucan	-	40 mg/kg BW	↓ fatty acid synthesis (SREBP-1c and FAS); adipogenesis (PPARγ); TC synthesis (HMG-CoA and FABP4)	Reduce body weight, TC, TG, LDL-c, and adiponectin levels; increase HDL-C and leptin levels	[116]

**Table 3:** Metabolic marker and gut microbiota regulation by plant polysaccharides in obese mice.

Polysaccharide Source	Mouse Model	Dose	Mechanism	Effects	Effect on Gut Microbiota	Key Structural Features	Reference
*Rosa roxburghii fruit* polysaccharides	C57BL/6J	200 mg/kg BW 400 mg/kg BW	Inhibit inflammatory signaling pathways (TLR4 and NF-κB p65); ↓mRNA expression	↓ body weight, TG, TC, LDL-C, and LDL-C/HDL-C ratio levels	↑ *Oscillospiraceae* and *Tannerellaceae*	*Mw* ~67.2 kDa; composed of arabinose (37.2%), galactose (34.4%), glucose (10.0%), fucose (18.3%); pectic backbone with mixed α/β linkages	[117,118]
Sea buckthorn polysaccharide	C57BL/6J	0.1% *w*/*w* SP of HFD	Activate the AMPK pathway	↓ body weight, TC, TG, LDL-c, ALT, and AST levels; ↑HDL-C levels	↑ abundance of *Muribaculaceae unclassified*, *Bifidobacterium*, *Rikenellaceae RC9*, *Alistipes*, and *Bacteroides*; ↓ *Lactobacillus*, *Firmicutes_unclassified*, *Dubosiella Bilophila*, and *Streptococcus*	*Mw* ≈6.26 × 10^3^ kDa; rich in galacturonic acid, galactose, rhamnose; irregular branched pectin–hemicellulose network	[73,119]
*Morchella esculenta* polysaccharides	C57BL/6J	100 mg/kg BW, 400 mg/kg BW	↓ mRNA levels of G6Pase, GLUT1, PPAR-α, PPAR-γ, and C/EBPα	↓ body weight, LDL-C, ALT, AST, ALP, and GGT levels; ↑ TNF-α, IL-1β, IL-6, and HDL-C levels	↓ *Firmicutes/Bacteroidetes* ratio; ↓ abundance of obesity-related *Faecalibaculum*; ↑abundance of *Dubosiella, Lactobacillus,* and *Rikenellaceae* RC9	Mw ~4.7 × 10^3^ Da (FMP-1) or ~3.0 × 10^3^ kDa (MMP-L); backbone of →4)-α-d-Glcp and →1,6/→4,6-linked Glc/Gal/Man units with branching	[120,121]
*Sargassum pallidum polysaccharide* polysaccharide	BALB/c	50 mg/kg BW, 100 mg/kg BW, 400 mg/kg BW	↓ TG synthesis and metabolism by decreasing mRNA levels of PPAR-γ, SREBP-1c, FAS, ACC1, and G6Pase	↓ body weight, TC, TG, LDL-c, GSH, and T-SOD levels; ↑ HDL-C levels		Fractions ~5.9–7.3 kDa; composition includes fucose, glucose, mannose, galactose, xylose	[122,123]
Kelp polysaccharide	C57BL/6J	300 mg/kg BW	↓ TG synthesis (SREBP-1c and FAS); decrease TC synthesis (HMGCR); ↑ TC consumption (CYP7A1 and CYP27A1)	↓ bodyweight, TC, TG, LDL-c, ALT, and AST; ↑ HDL-C levels	↓ *Colidextribacter* abundance; ↑ *Desulfovibrio* abundance	Typical sulfated α-l-fucose backbone (such as (1→3) and (1→4) linked); high sulfate ester content (≈30–40%); *Mw* range 50–1000 kDa	[124]
Barley grass polysaccharides	C57BL/6J	200 mg/kg BW 400 mg/kg BW	↓ TNF-α and IL-6 levels	↓ body weight, TC, TG, and LDL-C levels	↑ relative abundance of *Bacteroidetes*, *Bacteroidacea*, and *Lachnospiraceae*; ↓ *Firmicutes*/*Bacteroidetes* ratio and relative abundance of *Desulfovibrio*	Arabinoxylan-type heteropolysaccharide rich in arabinose/xylose; high insoluble fiber; *Mw* not specified but branched xylose backbone typical	[125,126]
*Caulerpa racemosa* sulfated polysaccharide	Rattus norvegicus	65 or 130 mg/kg BW/d orally for 8 weeks	Activates mTOR-SIRT1-AMPK and PRMT1-DDAH-ADMA pathways: upregulates SIRT1, AMPK, DDAH-II, PGC-1α, SOD; downregulates PRMT1, mTOR, TNF-α, HMGCR	↓ body weight gain; ↓ TG, LDL-C, TC, blood glucose; ↑ HDL; improved insulin sensitivity and reduced hepatic steatosis	↑ *Firmicutes/Bacteroidetes* ratio; SPCr reverses this and increases *Bacteroides*, *Parabacteroides*, *Alloprevotella*, *Ruminococcus*; ↓ *Desulfovibrionaceae*, *Bilophila*; lowers circulating LPS	Rhamnose and xylose-based sulfated backbone with side chains of mannose, arabinose, galactose;	[127]
*Artemisia sphaerocephala Krasch seed* polysaccharides (ASKP1, ASKP2, ASKP3)	C57BL/6J mice fed an HFD	400 mg/kg·bw	Modulation of gut microbiota and thermogenesis	Reduction in body weight, liver and epididymal white adipose tissue (eWAT) indices; improvement in glucose and lipid metabolism; elevation of antioxidant capacity; alleviation of inflammation	ASKP1 promotes the proliferation of beneficial bacterium *Akkermansia* more effectively than ASKP2 and ASKP3; increases the abundance of beneficial bacteria such as *Blautia*, *Christensenellaceae_R-7_group*, *Romboutsia*, and *Allobaculum*	ASKP1: Neutral heteropolysaccharide with an average molecular weight of 9.08 × 10^5^ Da; ASKP2 and ASKP3: Acidic heteropolysaccharides with molecular weights of 9.39 × 10^5^ and 8.41 × 10^5^ Da, respectively	[128]
*Ginseng* polysaccharide from ginseng root slices	C57BL/6J	100 mg/kg·bw	Inhibits hepatic lysine degradation (downregulates AASS, ALDH7A1, NSDHL); improves lipid metabolism and antioxidant capacity	↓ body weight, liver index, TG, TC, ALT, AST; ↑ SOD, T-AOC; improved liver histology	↑ *Lactobacillus, Bifidobacterium*, *Bacteroides*; ↓ *Firmicutes/Bacteroidetes* ratio; ↑ SCFA-producing bacteria	Predominantly composed of glucose (94.91%) primarily contains pyranose-type monosaccharides; glycosidic linkages are primarily in α-configuration	[129]
*Auricularia auricula-judae (Bull.)* polysaccharides	C57BL/6J	50 mg/kg·bw	Modulates TLR4/JNK; activates AMPK/AKT; improves gut barrier; increased SCFAs	↓ body weight; ↓ lipid accumulation; ↓ inflammation; ↑ SCFAs; improved metabolic profile	↑ SCFA-producing bacteria; ↓ harmful bacteria	*Mw* ≈ 1.21 × 10^6^ Da; 67.68% neutral sugar, 25.50% uronic acids; rich in mannose, galacturonic acid; β-pyranose (1069, 905 cm^−1^)	[130]
*Psidium guajava crude* polysaccharides	C57BL/6J	100 mg/kg·bw	Modulates gut microbiota; regulates TLR4/JNK signaling pathway; enhances SCFA production	↓ body weight gain; ↓ visceral obesity; ↓ serum cholesterol, TG, LDL-C; ↓ liver lipid accumulation; improved insulin resistance and liver inflammation	↑ *Clostridium XlVa*, *Parvibacter*, *Enterorhabdus*; ↑ SCFAs (primarily butyrate); restored *Firmicutes/Bacteroidetes* ratio; ↓ *Mucispirillum*	*Mw* of 8.0 × 10^5^ and 2.2 × 10^4^, Galacturonic acid, Galactose, Arabinose in a molar ratio of 3:1:6	[131]
Polysaccharide fraction from *Raphanus sativus greens*	C57BL/6J mice	4 mg/kg BW (oral gavage)	Improves gut barrier integrity, modulates gut microbiota, suppresses expression of lipid metabolism-related genes	↓ Body weight gain; ↓ visceral fat mass; ↓ adipocyte size; ↓ serum TC, TG, and LDL-C levels	Restored *Firmicutes/Bacteroidetes* ratio; shifted gut microbiota toward a healthier profile	*Mw*: 61.1 kDa; *Mn*: 3.91 kDa; *Mw*/*Mn* = 15.6; composed of 70.8% neutral sugar and 22.3% uronic acid; dominant monosaccharides: galactose (40.7%), arabinose (22.9%), galacturonic acid (12.7%)	[132]

**Table 4:** Summary of clinical trials involving plant polysaccharides.

Polysaccharide Source	Subjects	Dose	Key Outcomes	Reference
Oat β-glucan	19 healthy adults	2–4 g/30 g carbs, acute and postprandial (lunch) meals	↓ peak glucose iPeak (*p* < 0.05); ↓ iAUC (0–60 min, *p* < 0.05); ↓ insulin iAUC (*p* < 0.05); increased satiety	[133]
Oat β-glucan	35-trial meta-analysis	Median 2.8 g/30 g carbs	↓ postprandial glucose iAUC and insulin iAUC; effect modified by *M_W_*	[134]
Barley β-glucan	Hypercholesterolemic/overweight	3–10 g/day, 4–12 week	↓ LDL-C, TC; improved glycemia	[135]
Inulin	40 overweight adults	10 g/day, 6 weeks	↓ weight; ↓ insulin; ↑ GLP-1; ↑ probiotic content	[136]
Arabinoxylan (wheat bran)	Overweight adults	Varies, RCT	↑ insulin sensitivity; ↓ LDL	[137]
Psyllium husk	T2DM adults	Not specified, meta-analysis	↓ FBS, HbA1c, HOMA-IR; improved glycemic control	[138]
Konjac glucomannan	Overweight adults	3.99 g/d, 8 weeks	↓ BMI, weight; improved satiety; well tolerated	[139]
Pectin (apple)	Obese adults	15 g/meal	↑ satiety; delayed gastric emptying	[140]

## Data Availability

No data was used for the research described in the article.

## Abbreviations

T2DM	Type 2 diabetes mellitus
JNK	Jun N-terminal kinase
IKK	IκB kinase
SCFAs	Short-chain fatty acids
WAT	White adipose tissue
BAT	Brown adipose tissue
UCP1	Uncoupling protein 1
AMPK	AMP-activated protein kinase
PPAR-α	Peroxisome proliferator-activated receptor α
PPAR-γ	Peroxisome proliferator-activated receptor γ
SREBP-1c	Sterol regulatory element-binding protein 1c
FASN	Fatty acid synthase
ACC	Acetyl-CoA carboxylase
LEP	Leptin
CCK	Cholecystokinin
GLP-1	Glucagon-like peptide-1
PYY3-36	Peptide YY 3-36
α-MSH	Alpha-melanocyte-stimulating hormone
TNF-α	Tumor necrosis factor α
IL-1	Interleukin-1
IL-6	Interleukin-6
iNOS	Inducible nitric oxide synthase
MCP-1	Monocyte chemoattractant protein-1
TGR5	G protein-coupled bile acid receptor 1
FXR	Farnesoid X receptor
F/B	Firmicutes/bacteroidetes ratio
GPR43	G protein-coupled receptor 43
GPR41	G protein-coupled receptor 41
BAs	Bile acids
CYP7A1	Cholesterol 7α-hydroxylase
CYP27A1	Cholesterol 27-ydroxylase
CYP7B1	Cholesterol 7β-hydroxylase
SHP	Small heterodimer partner
FGF15	Fibroblast growth factor 15
POMC	Pro-opiomelanocortin
MC4Rs	Melanocortin 4 receptor
LPBN	Lateral parabrachial nucleus
